# Supplementary material for: CD301b+ Macrophages as Potential Target to Improve Orthodontic Treatment under Mild Inflammation
Source: Cells. 2022 Dec 29;12(1):135. doi: 10.3390/cells12010135 (PMC9818444; doi:10.3390/cells12010135)
Supplement: Supplementary file 1 [file cells-12-00135-s001.zip › cells-2095884-supplementary.pdf]

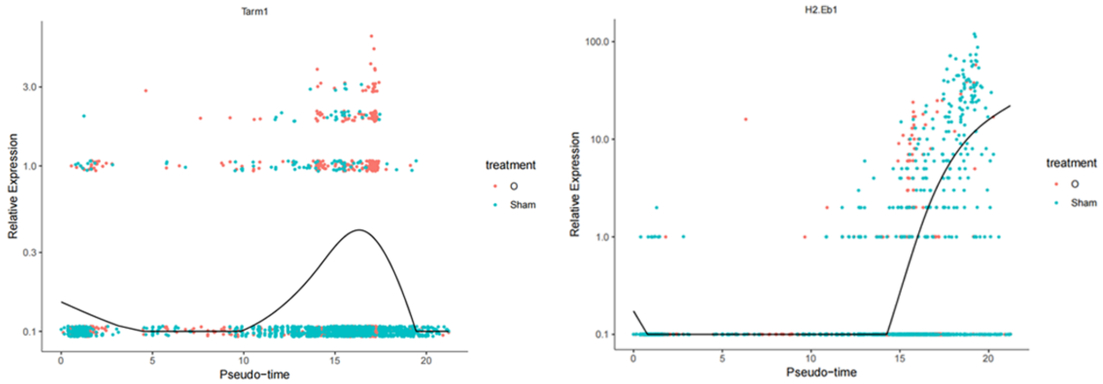

Figure S1: Single cell trace analysis of different genes was performed using Monocle.

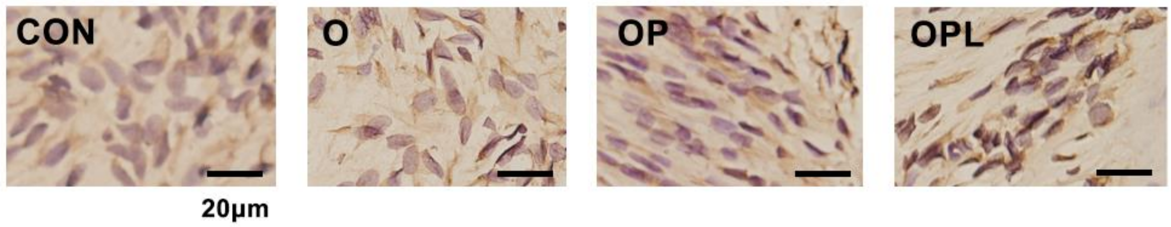

Figure S2: Expression of immunohistochemical inflammatory marker TNF-α (scale bar = 20 μ m) .

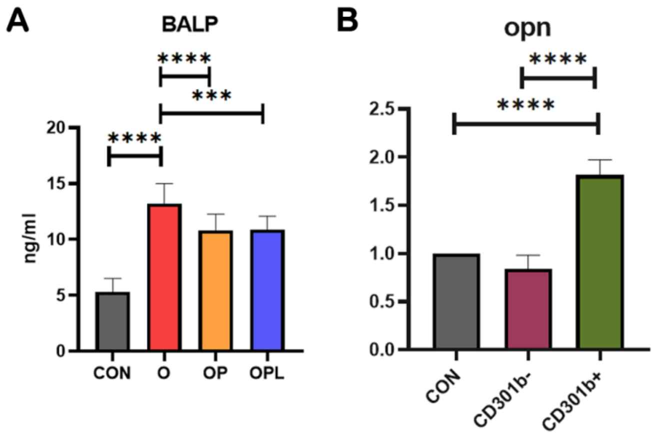

Figure S3: (A)Expression of bone metabolic marker BALP. (B)Expression of osteogenesis-related gene OPN in BMSC under stretching force after osteogenesis induction was detected using qRT-PCR. Values are presented as mean ± SD. ***$P < 0.001$, ****$P < 0.000\ 1$. n=3-6.

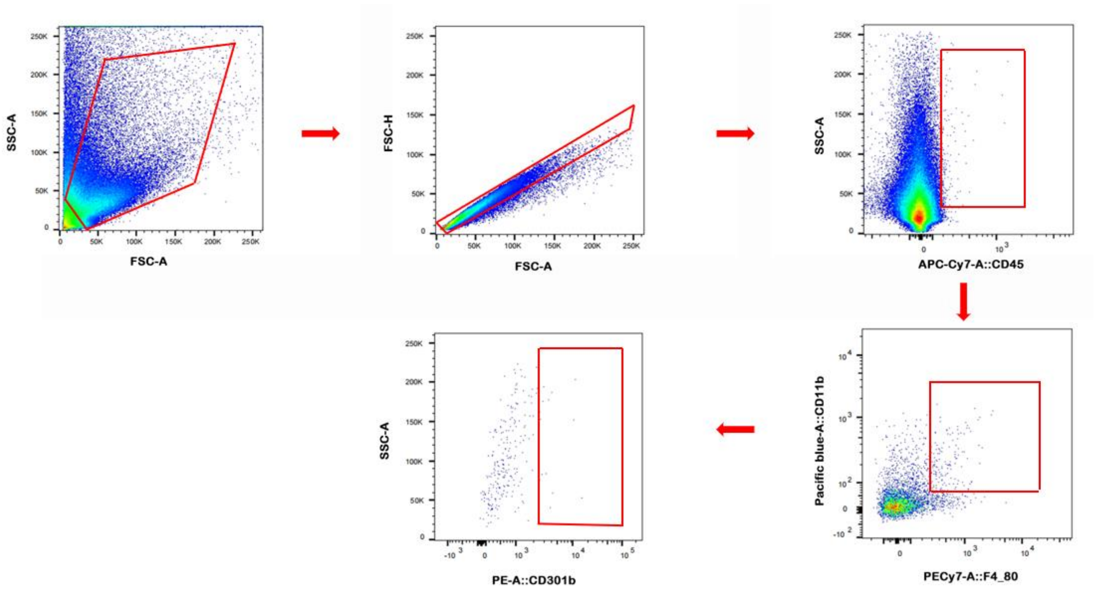

Figure S4: Gating strategy of CD301b+ macrophages.

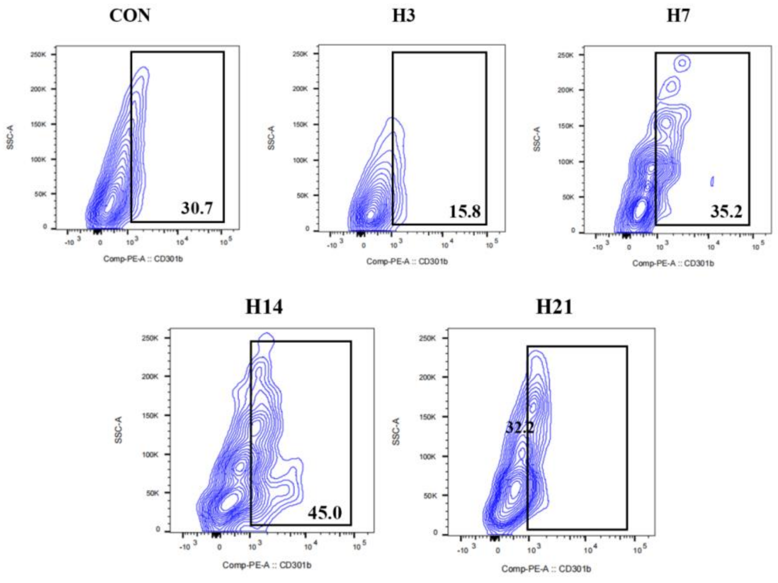

Figure S5: Flow cytometry plots of CD301b+ macrophage content around orthodontically mobile teeth in orthodontic models after 3, 7, 14, and 21 days of periodontitis unlining in control and periodontal models.
